# A Case of Sub-occlusive Free-Floating Thrombus in the Basilar Artery Causing Stroke-Like Symptoms: A Case Report

**DOI:** 10.7759/cureus.91466

**Published:** 2025-09-02

**Authors:** Justin Kadamala Samuel, Arun Cherackakudy Joy, James Beckett, Balakrishna Kumar, Riaz Ali

**Affiliations:** 1 Department of Internal Medicine, Portsmouth Hospital University, Portsmouth, GBR; 2 Department of General Medicine, Portsmouth Hospital University, Portsmouth, GBR; 3 Department of Stroke, Portsmouth Hospital University, Portsmouth, GBR; 4 Department of Radiology, Portsmouth Hospital University, Portsmouth, GBR

**Keywords:** and transient ischemic attack (tia), basilar stroke, brain thrombectomy, fenestrated basilar artery, free floating thrombus, stroke, tia mimics

## Abstract

Free‑floating thrombus (FFT) - also known as intraluminal thrombus - refers to a clot that remains partially adherent to the vessel wall yet projects into the lumen, permitting movement with the bloodstream. Since its attachment is tenuous, the thrombus carries a heightened risk of fragmentation, embolization, or partial obstruction of blood flow, thereby significantly increasing the likelihood of developing a transient ischemic attack or ischemic stroke. The patient may exhibit motor, sensory, and/or cortical symptoms. Regardless of the severity, immediate intervention can lead to a complete neurological recovery.

In this case report, we present the scenario of a 28-year-old female patient with symptomatic sub-occlusive basilar artery thrombus, who developed stroke-like symptoms, subsequently undergoing multiple investigations and interventions, including thrombolysis and thrombectomy. Identifying a sub‑occlusive basilar artery thrombus in a patient exhibiting classic stroke symptoms, but without any ischemic changes evident on imaging, is considered highly unusual and represents a rare diagnostic challenge. This case reveals that FFT should be considered in the differential diagnosis of patients presenting with acute neurological symptoms, even in the absence of typical ischemic changes on initial imaging. Early diagnosis and aggressive management with high-dose dual antiplatelet therapy (Aspirin 300 mg and Clopidogrel 300 mg as loading doses) in addition to thrombolysis and mechanical thrombectomy can lead to favorable outcomes. This case highlights the value of a multidisciplinary strategy and underscores the urgent need for further research to clarify the pathophysiology and optimize management of FFT.

## Introduction

A free-floating thrombus (FFT) can be best defined as an elongated thrombus attached to the arterial wall with circumferential blood flow at its distal-most aspect with cyclical motion relating to cardiac cycles [1]. FFT is most commonly reported in the internal carotid artery; intracranial involvement is exceedingly rare, with posterior circulation cases occurring in only about 0.3%-0.5% of ischemic strokes. Reports of FFT affecting the basilar artery are notably scarce, presenting significant challenges in both diagnosis and management. This case report of FFT in the basilar artery serves to underscore the diagnostic difficulties and therapeutic decision-making challenges posed by this rare presentation.

Etiology and clinical presentation

The precise pathogenesis of FFT remains elusive, with hypercoagulability and atherosclerosis emerging as plausible etiologies [2]. Less common causes encompass arterial dissection and substance abuse [3,4]. WhileFFT may remain asymptomatic and incidentally detected, symptomatic cases typically present with acute neurological deficits stemming from cerebral ischemia, ranging from transient ischemic attacks (TIAs) to established infarction [5]. In the case of Basilar artery thrombosis, the prodromal phase often exhibits a prolonged latency period, characterized by nonspecific symptoms such as nausea, vomiting, dizziness, etc. [6]. As the FFT progresses, more pronounced neurological deficits may surface, including diplopia, dysarthria, dysphagia, altered consciousness, and motor/sensory impairments.

Diagnostic challenges and imaging modalities

The diagnostic journey of FFT is intricate due to the absence of standardized radiological features. Imaging techniques like magnetic resonance imaging (MRI), magnetic resonance angiography (MRA), and computed tomography angiography (CTA) play a pivotal role in visualization. Digital subtraction angiography (DSA) stands as the gold standard for detailed vascular imaging, albeit invasive [7].

Therapeutic strategies

Managing FFT necessitates a comprehensive approach targeting both the thrombus and its underlying cause. Therapeutic modalities encompass anticoagulation therapy, thrombolytic therapy, and endovascular interventions like mechanical thrombectomy or aspiration for thrombus removal [1]. Each therapeutic avenue carries distinct risks and benefits, mandating a personalized and multidisciplinary care strategy.

## Case presentation

A 28-year-old right-handed woman presented to the emergency department with a history of almost a week-long history of dizziness, blurred vision, nausea, vomiting, and a sudden-onset headache, described as a severe frontal headache rated at 8/10, which was unrelieved by medications.

Background and medical history

The patient had a history of migraine, recurrent first-trimester miscarriages (three episodes), and three syncopal episodes within the last month. Prior comprehensive evaluation for hypercoagulability, including assessments for lupus anticoagulant and thrombophilia panels, yielded unremarkable findings.

Clinical examination

On examination, the patient’s vital signs were stable: blood pressure 107/79 mmHg, pulse 86 bpm, afebrile, respiratory rate 16/min, and oxygen saturation 98% on room air. Neurologically, the patient had a Glasgow Coma Scale (GCS) score of 15/15 and exhibited posterior circulation stroke symptoms like partial gaze palsy, facial paralysis, dysarthria, dysphagia, with an NIH Stroke Scale (NIHSS) score of 14. There was no truncal ataxia, tremors, cognitive impairment, cerebellar dysfunction, or abnormal gait. 

Diagnostic imaging and findings

**Figure 1:**
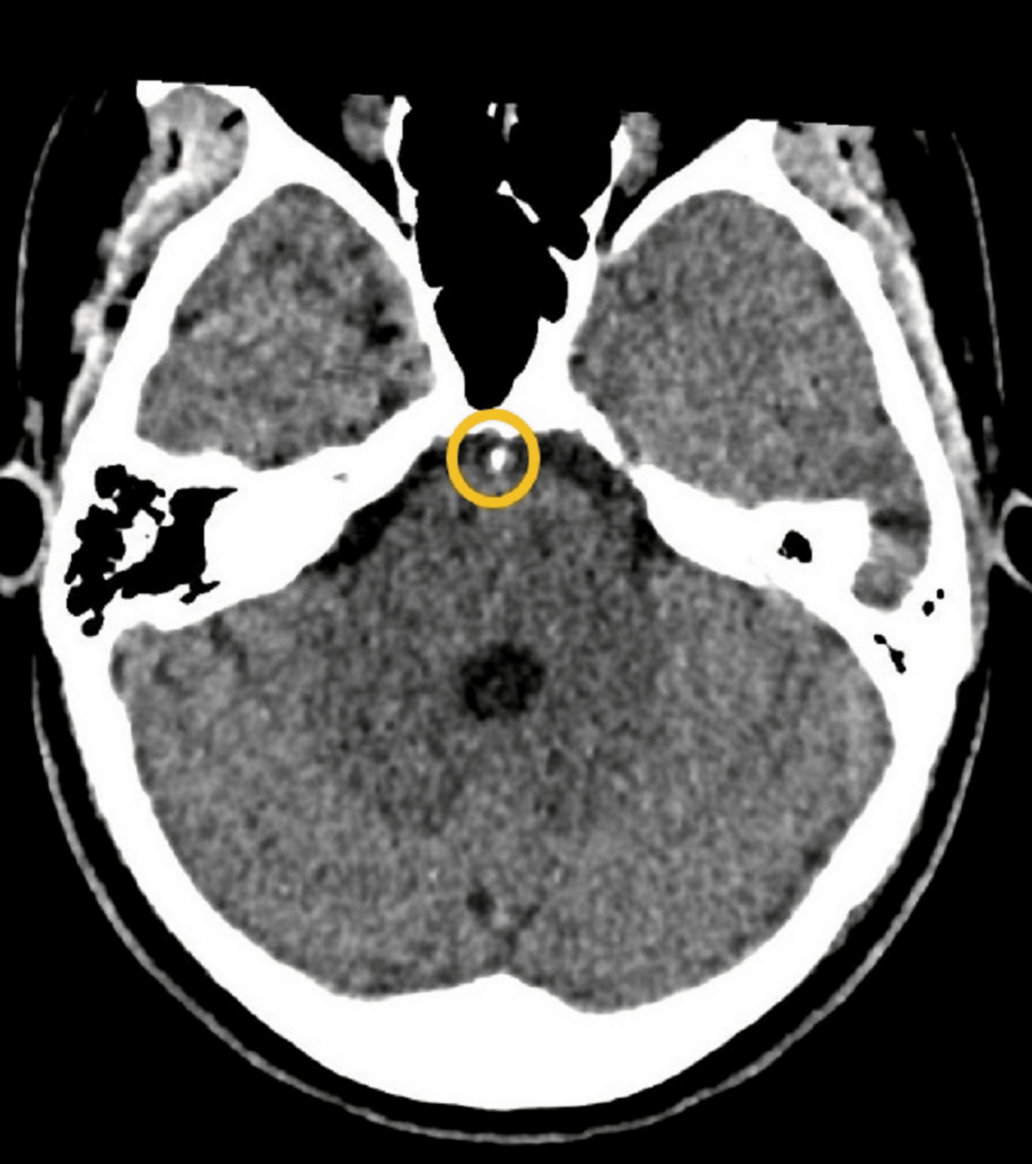
CT head, unenhanced: Axial image showing a focal hyperdensity (mean Hounsfield unit = 150) at the level of the mid-basilar artery, thought to represent an atherosclerotic plaque/calcification or possibly a small thrombus.

**Figure 2:**
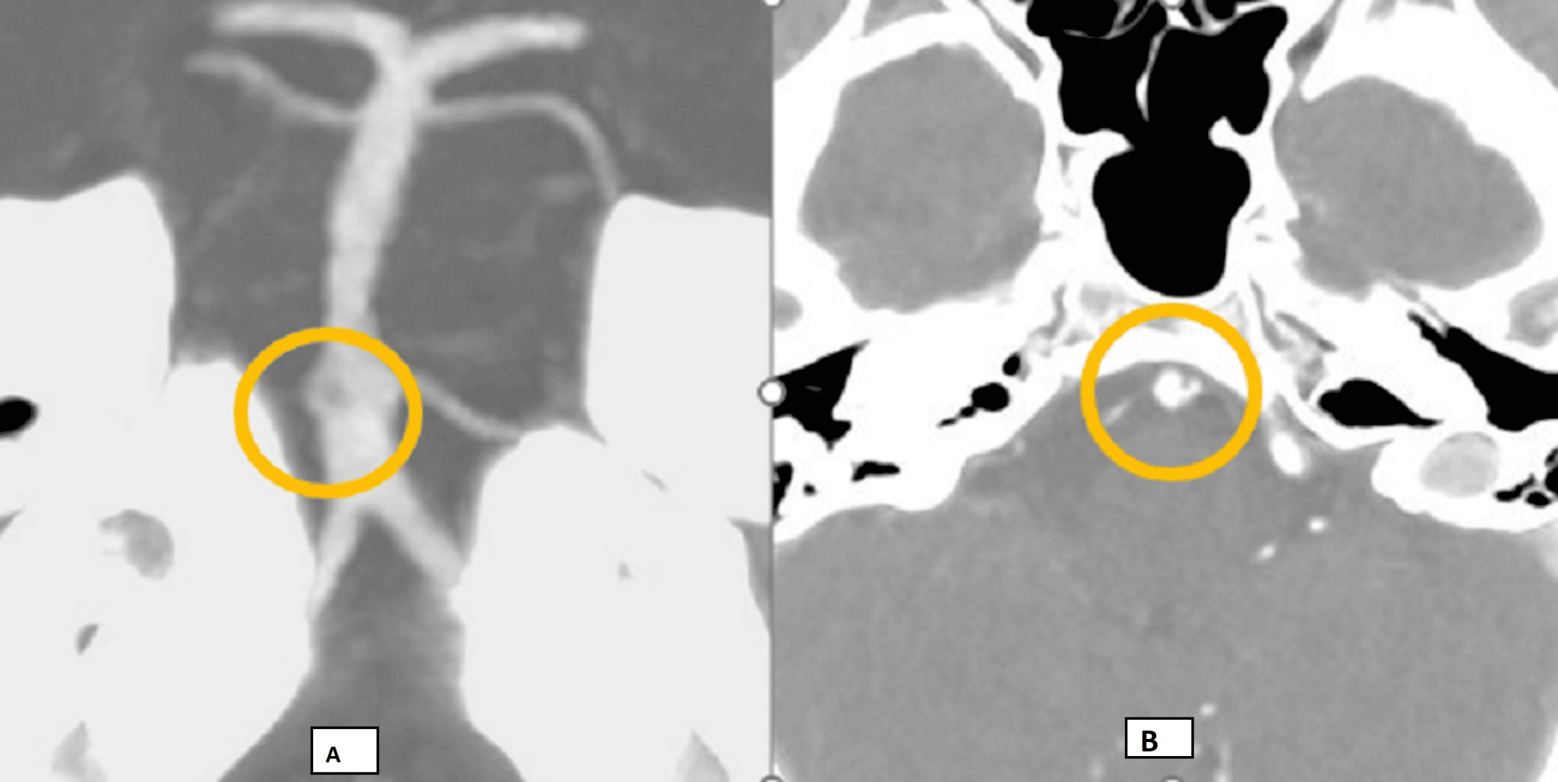
(A) CT angiogram, coronal maximum intensity projection (MIP), showing no abnormality at the level of the mid-basilar artery, although an incidental flap was noted at the proximal basilar artery (at the level of the AICAs), thought to represent a dissection; (B) axial image showing the suspected dissection of the proximal basilar artery. AICA, anterior inferior cerebellar artery

The stroke team was alerted, and an urgent CT brain was done. It showed no ischemic changes but revealed an area of focal hyperdensity at the level of the mid-basilar artery, thought to represent an atherosclerotic plaque/calcification or possibly a small thrombus (Figure 1), a finding atypical for the patient’s young age. Further assessment included a CT angiogram, which revealed no abnormality at the level of the mid-basilar artery, but an incidental flap was noted at the proximal basilar artery, which was initially reported as possible dissection (Figures 2A, 2B).

**Figure 3:**
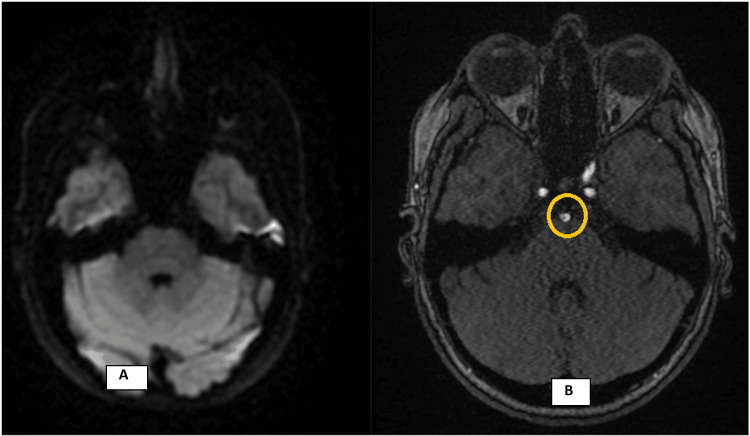
(A) MRI DWI b1000 image showing no diffusion changes in the posterior fossa; (B) TOF-MRA demonstrating a small central signal void in the mid-basilar artery, corresponding to the site of the hyperdensity previously seen on CT. TOF-MRA, time-of-flight magnetic resonance angiography; MRI DWI, magnetic resonance imaging with diffusion-weighted imaging

Given the unclear and inconsistent CT findings, an MRI/MRA (Figure 3) was performed on the day after the initial presentation, which revealed that the incidental flap noted in the CT at the basilar artery was, in fact, a fenestration. It also identified a flow void in the time-of-flight (TOF) sequences, which was later identified to be an FFT. Again, no ischemic changes were observed at this stage. The patient also underwent a lumbar puncture, which showed an elevated white cell count, leading to empirical treatment with antibiotics to cover for possible encephalitis, but the cerebrospinal fluid (CSF) culture did not reveal any bacterial growth.

**Figure 4:**
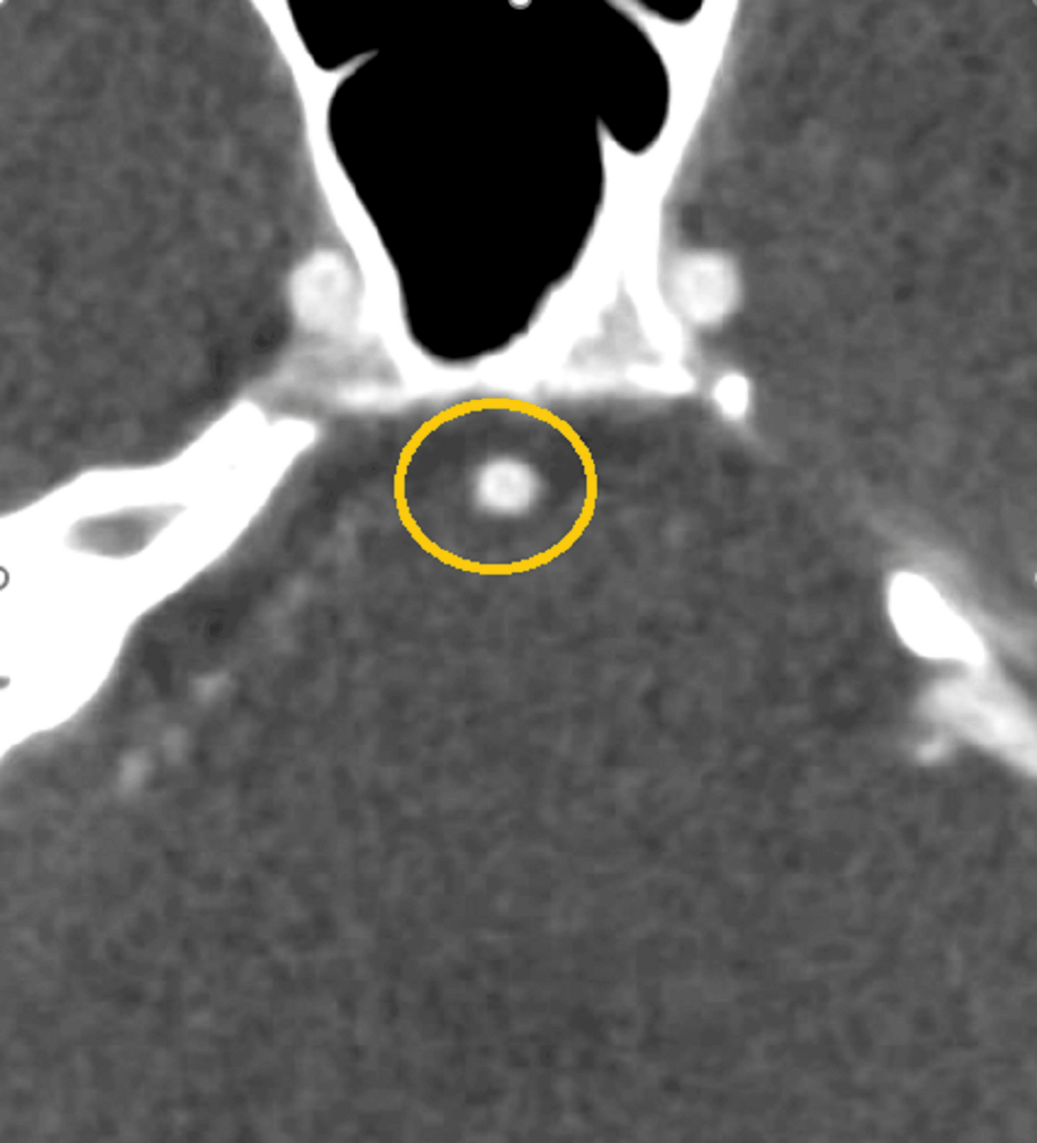
CT angiogram, axial image at the level of the mid-basilar artery, retrospectively showing a possible central filling defect (donut sign).

**Figure 5:**
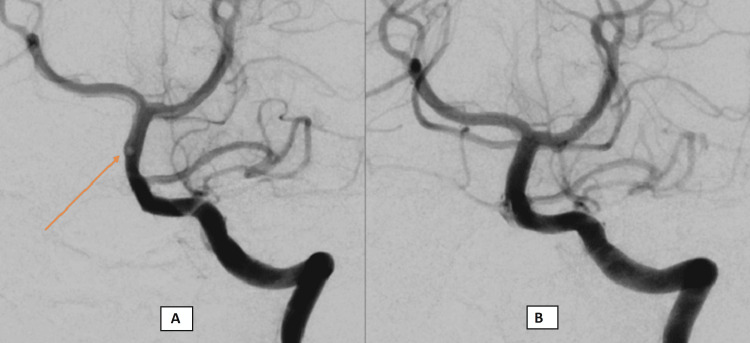
(A) DSA anteroposterior view showing a free-floating thrombus in the right side of the mid-basilar artery; (B) post-thrombectomy image demonstrating successful clot retrieval. The proximal basilar *flap* at the level of the AICAs was confirmed to represent a normal variant fenestration rather than a dissection. AICA, anterior inferior cerebellar artery; DSA, digital subtraction angiography

A few hours later, on the same day, she developed new neurological symptoms, including an unsteady gait, right-sided facial droop, limb weakness, and reduced right-sided sensation. An emergency repeat CT brain angiogram was done, which revealed a possible central filling defect at the level of the mid-basilar artery *segment* resembling the *donut sign* (Figure 4), which is an image marker of intraluminal thrombus. Since the evidence was inconclusive, the patient underwent an urgent digital subtraction angiography (DSA), which confirmed an FFT in the right side of the mid-basilar artery (Figure 5). Due to the recurrent and worsening neurological symptoms, the patient was diagnosed to have a symptomatic basilar artery thrombosis presenting as a TIA with a high risk of imminent stroke. As the patient was still in the therapeutic window, a decision to thrombolyze the patient was made. The patient was thrombolyzed using Alteplase. 

Treatment and outcome following thrombolysis

Post-thrombolysis, the patient deteriorated within 12 hours, developing ophthalmoplegia and quadriparesis, with an NIHSS score of 20. Because of the rapid progression of her neurological symptoms despite thrombolysis, an urgent mechanical thrombectomy was performed using the aspiration thrombectomy technique, which successfully retrieved the thrombus from the mid-segment of the basilar artery (Figure 5). A follow-up MRI post-procedure showed no ischemic damage, and the patient's NIHSS score improved to 2. The patient subsequently made a near full neurological recovery, with a Modified Rankin Scale score of 1, and was discharged home. When the patient was followed up as an outpatient a few weeks later, her neurological symptoms had fully resolved, with no functional disability (Modified Rankin Scale score of zero). She underwent an extensive workup, including a repeat hemophilia screen, echocardiogram (ECHO), 12-lead electrocardiogram (ECG), and 72-hour ambulatory ECG monitoring, all of which revealed no abnormalities. Serial MRI scans done later confirmed the absence of an ischemic insult.

## Discussion

FFT occurring in the posterior circulation is associated with higher mortality rates compared to an FFT in the carotid territory [8]. FFT is increasingly recognized as an important imaging marker for ischemic stroke [9,10], despite limited reports on asymptomatic cases.

This case highlights two key lessons: the patient’s unique clinical presentation and the importance of choosing the correct management strategy. FFT is often seen in middle-aged men  [1] and is most commonly linked to atherosclerosis and hypercoagulability [1,2]. Our patient was a young woman with low conventional risk factors for atherosclerosis. Her history of recurrent miscarriages and syncopal episodes could suggest a potential underlying vascular or hypercoagulable condition.

The presence of fenestration in the basilar artery could also have contributed to the patient developing FFTs. Fenestrations are more commonly observed in the posterior circulation (73.2%) compared to the anterior circulation (24.6%) [11]. A pilot study done by Dong et al.  [12] indicated that the altered flow dynamics in basilar artery fenestrations could heighten the risk of thrombus formation, plaque instability, and subsequent ischemic events. Another case, as noted by Berry et al. [13], involved a 43-year-old man who died from a large pontine infarct due to a partially occluded basilar artery, likely caused by hemodynamic disturbances at a fenestration site.

FFT usually presents with acute neurological deficits. When A Buchan et. al evaluated 30 patients with intraluminal thrombus, they found that stroke was the presentation in 22 patients (12 had previous TIAs), TIAs occurred alone in seven cases, and one patient was asymptomatic [8]. A 2015 study by Organek et al. [14]​​​​documented patients with mild, transient symptoms like vertigo, nausea, headache, and neck pain, occurring about two weeks before severe exacerbation [15]. Patients with FFT often exhibit acute and fluctuating neurological symptoms due to transient vessel obstruction by the clot or artery-to-artery embolism, leading to sudden deficits, which could explain the waxing and waning neurological symptoms seen in our patient. A lack of standardized diagnostic criteria makes diagnosing an FFT challenging. The main radiological sign seen in FFT is the donut sign on computed tomography angiography, representing a thrombus as a filling defect surrounded by contrast [14]. DSA is considered the gold standard for diagnosing mural thrombus [16]. In this case, the *donut sign* in the basilar artery on CTA and the identification of a basilar artery mural thrombus on DSA provided definitive confirmation of an FFT.

Both medical and surgical interventions have been used in managing FFTs. Medical management involves anticoagulation and antiplatelet therapy, and surgical management options include stenting, bypass, endarterectomy, or endovascular treatment [8]. While surgical intervention is common (68% of cases, as per Bhatti et al. [1]), approximately 30% of FFT cases are managed with medications, yielding comparable outcomes at 30 days. However, no randomized trial exists to support the comparison of medical versus surgical treatment. Therefore, treatment options are often chosen based on individual clinical scenarios. Medical management is often considered the first-line treatment because of the risks associated with surgical measures like bleeding or embolization. In the case of our patient, her presentation, along with the lack of ischemic changes in her CT or MRI, made diagnosing and choosing the right treatment modality very challenging. The patient was initially managed medically, but as her condition worsened, with the patient developing progressively worsening neurological findings, a decision was made to pursue surgical treatment, despite the risks. As evidenced by Roy et al. [16], patients with FFT can have recurrent TIAs before developing an acute stroke; thus, making early intervention very critical. FFTs carry a significant short-term risk of progression to stroke, up to a 17.1% rate of TIA, silent ischemia, stroke, or death within 30 days [9], which should also be taken into account while deciding the treatment strategy. Our patient underwent thrombolysis, followed by urgent mechanical thrombectomy with clot retrieval, resulting in complete resolution of symptoms. 

## Conclusions

This case underscores the intricate nature of diagnosing and treating cerebrovascular anomalies, such as FFTs. Because FFTs carry a high risk of recurrent stroke or TIAs, rapid detection and intervention are essential. However, the absence of formal guidelines for managing FFTs makes handling complex cases like ours particularly challenging.

Both medical and surgical interventions have been employed in the management of symptomatic FFTs, but there is currently no consensus on which approach is optimal. In our patient, who experienced recurrent neurological events without definitive infarction, the treatment plan necessitated a nuanced risk-benefit assessment tailored to the individual clinical context. Given that FFTs may initially present solely with TIAs and carry a significant risk of evolving into stroke, we opted for early, assertive intervention to maximize the chance of complete neurological recovery, despite the absence of radiologically confirmed ischemia.
